# Framework and approach for measuring performance and progress of mental health systems and services in India: National Mental Health Survey 2015–2016

**DOI:** 10.1186/s13033-020-00349-8

**Published:** 2020-03-13

**Authors:** Banavaram Anniappan Arvind, Gopalkrishna Gururaj, Girish N. Rao, Banandur S. Pradeep, Varghese Mathew, Vivek Benegal, Melur Sukumar Gautham, Amudhan Senthil, Arun Kokane, Bir Singh Chavan, P. K. Dalal, Daya Ram, Kangkan Pathak, Singh R. K. Lenin, Lokesh Kumar Singh, Pradeep Sharma, Pradeep Kumar Saha, C. Ramasubramaniam, Ritambhara Y. Mehta, T. M. Shibukumar, the NMHS collaborators group, Lokesh Kumar Singh, Sonia Pereira Deuri, Mousumi Krishnatreya, Vijay Gogoi, H. Sobhana, Saumik Sengupta, Indrajeet Banerjee, Sameer Sharma, Anjan Kumar Giri, Abhay Bhaskar Kavishvar, Kamlesh Rushikray Dave, Naresh T. Chauhan, Vinod K. Sinha, Nishanth Goyal, Jayakrishnan Thavody, P. K. Anish, Thomas Bina, Abhijit P. Pakhare, Pankaj Mittal, Sukanya Ray, Rajni Chatterji, Brogen Singh Akoijam, Heramani Singh, Gojendro, Priscilla Kayina, L. Roshan Singh, Subhash Das, Sonia Puri, Rohit Garg, Amita Kashyap, Yogesh Satija, Kusum Gaur, Divya Sharma, R. V. Sathish, M. Selvi, Krishnaraj, S. K. Singh, Vivek Agarwal, Eesha Sharma, Sujit K. Kar, Raghunath Misra, Rajashri Neogi, Debasish Sinha, Soumyadeep Saha, Ajoy Halder, D. K. Subbakrishna, Thennaarasu, P. Marimuthu, B. Binu Kumar, Sanjeev Jain, Santosh Loganathan, Y. C. Janardhan Reddy, T. Jagadisha, P. T. Sivakumar, Prabhat Kumar Chand, K. Muralidharan, Senthil Reddi, C. Naveen Kumar, M. Krishna Prasad, T. S. Jaisoorya, C. N. Janardhanan, Mahendra Prakash Sharma, L. N. Suman, S. Paulomi, Keshav Kumar, Manoj Kumar Sharma, M. Manjula, Poornima Bhola, B. N. Roopesh, M. Thomas Kishore, S. Veena, K. Aruna Rose Mary, Nitin Anand, Shobha Srinath, Satish Chandra Girimaji, K. John Vijayasagar, Sekar Kasi, D. Muralidhar, R. Dhanasekara Pandian, Ameer Hamza, N. Janardhana, E. Aravinda Raj, Gobinda Majhi

**Affiliations:** 1 Centre for Public Health, Department of Epidemiology, National Institute of Mental Health and Neuro Sciences, Bangalore, 560029 India; 2 Department of Psychiatry, National Institute of Mental Health and Nero Sciences, Bangalore, 560029 India; 3 Department of Community Medicine, All India Institute of Medical Sciences, Bhopal, India; 4 Department of Psychiatry, Government Medical College and Hospital, Chandigarh, India; 5 Department of Psychiatry, King George’s Medical University, Lucknow, India; 6 Department of Psychiatry, Central Institute of Psychiatry, Ranchi, India; 7 Department of Psychiatry, LGB Regional Institute of Mental Health, Tezpur, India; 8 Department of Psychiatry, Regional Institute of Medical Sciences, Imphal, India; 9 Department of Psychiatry, All India Institute of Medical Sciences, Raipur, India; 10 Department of Psychiatry, Sawai Man Singh Medical College, Jaipur, India; 11 Department of Psychiatry, Institute of Psychiatry, Kolkata, India; 12 Mental Health Program Office, Chennai, Tamil Nadu India; 13 Department of Psychiatry, Government Medical College, Surat, India; 14 Department of Psychiatry, Institute of Mental Health and Nero Sciences, Kozhikode, India

**Keywords:** Mental Health systems, Services, Methodology, Performance, Progress, India

## Abstract

**Background:**

Previous attempts of Mental Health Systems Assessment in India were restricted in scope and scale. Information on all aspects of mental health systems (leadership/governance, legislation, financing, service delivery, workforce, access to essential medicines, information systems, intersectoral activities, and monitoring and evaluation) was scarcely available. The National Mental Health Survey-Mental Health Systems Assessment (NMHS-MHSA), a unique endeavor, assessed the performance of mental health systems and services through health systems assessment framework. The present paper discusses the design and methodology adopted under NMHS-MHSA along with emphasizing its implication for India and other LMICs.

**Methods:**

NMHS-MHSA was undertaken in 12 Indian states by contextually adapting WHO-AIMS instrument. Data was collated from several secondary sources including interviews of key stakeholders. Utilizing the data a set of 15-quantitative, 5-morbidity and 10-qualitative indicators were developed to summarize the functional status of mental health systems in the surveyed states. This information was authenticated through state level stakeholder’s consultation and consensus building workshops following which a state mental health systems report card with indicators was developed.

**Conclusion:**

The process and robust method of data compilation enabled NMHS-MHSA to be a reliable and comprehensive method for assessing mental health systems at the state level. It’s envisaged that the assessment provides requisite impetus for strengthening mental health program and mental health systems in India. Being less resource intensive, low -and middle- income countries can adopt NMHS-MHSA tool and methodology to assess their mental health systems with contextual modifications.

## Background

Mental, Neurological and Substance Use Disorders are major public health problems. They account for 11.1% of global DALYs (disability adjusted life years) and Depressive Disorders are 3^rd^ leading cause of disability [1, 2]. The burden of mental and substance use disorders (MSUDs) are relatively high in Low-and Middle-Income Countries (LMICs). A third of global MSUDs related DALYs are contributed by India and China alone [3]. The recently conducted National Mental Health Survey (NMHS) of India reported, that at any given time, 1 in every 10 adults needs care for one or more mental disorders [4].

Functional health system is vital for delivery of effective health services and they contribute to reducing the burden of mental disorders in the population. Quality care is essential for improving the quality of life of those affected by mental disorders [5]. High burden of mental disorders in India [4] emphasizes the need for comprehensive, integrated and well functioning mental health systems. However, health systems in LMICs are challenged with scarcity of resources, inequitable distribution and inefficient utilization [6]. Mental health systems in LMICs including India are poorly funded with substantially inadequate manpower and infrastructure for mental health [7, 8]. These factors along with stigma, discrimination and low level of mental health literacy contributes to high treatment gap for mental disorders in India [9] varying between 70%-92% [4].

Understanding and appraising mental health systems and services is essential for developing sound mental health policy, program and services [10]. Inorder to support development of such policies and programs, mental health systems assessment (MHSA) should consider appraisal of all the following essential building blocks of health system viz service delivery, health workforce, health information system, access to essential medicines, financing and leadership/governance [11]. However, previous attempts of MHSA in India were restricted in scope, as they either evaluated/examined service delivered under the National Mental Health Programme (NMHP)/District Mental Health Programme (DMHP) or assessed standards of care available in mental health care facilities [4, 12–17]. Consequently, there has been limited understanding regarding the status of mental health systems in India from a public health perspective.

### Rationale for NMHS-MHSA

The National Mental Health Survey of India [18] implemented in 2015-16 aimed to estimate the prevalence and assess pattern of MSUDs from a representative national sample drawn from 12 states of India along with identifying the treatment gap, disabilities and socioeconomic impact associated with MSUDs. In order to facilitate and strengthen development of comprehensive and integrated mental health systems for delivery of programs and services at the national and state levels, there was a need to simultaneously assess the nature and performance of mental systems in NMHS surveyed states [19]. Hence NMHS-MHSA was simultaneously implemented along with NMHS in the 12 states of India with the following objectives (i) to examine the available health and health related resources for mental health activities/programmes in the surveyed states, (ii) to examine the status of mental health services and programmes in the surveyed states through a systems assessment framework. Details of design and methodology of NMHS is published elsewhere [4, 20]. The present paper describes the need, design, methods and the process implemented under NMHS-MHSA.

### Framework of NMHS-MHSA

Considering the essential building blocks of health systems [11], NMHS-MHSA assessed the following 10 components of mental health system viz (1) Mental Health Policy and Mental Health action plan, (2) Infrastructure for the delivery of mental health care, (3) Health human resources for mental health from health and health related sectors (4) Delivery of mental health care including availability of psychotropic drugs in district hospitals/Community Health Centers (CHCs)/taluka hospitals/Primary Health Centers (PHCs) along with availability of follow up care/domiciliary care in the community and outreach activities for mental health problems, (5) Implementation of legislations for mental health, 6) Public education and IEC activities, (7) Linkages with other sectors/departments like education, women and child welfare, social welfare etc. (8) Budget for mental health activities, (9) Engagement with civil society, and 10) Programme monitoring—evaluation and research including monitoring of the quality/type/nature of services provided.

Comprehensive assessment of the mental health system involves coordination, collection and compilation of data from various sectors and sources including those from outside the formal health sector which provide mental health care services and support. Hence, it involves an iterative process of checking, triangulating and validating the collected data. These issues were meticulously considered within the framework of NMHS-MHSA.

## Methodology

### Preparatory phase

Two preliminary efforts undertaken by Centre for Public Health (http://nimhans.ac.in/center-for-public-health/), Department of Epidemiology @ National Institute of Mental Health and Neuro Sciences (NIMHANS), served as pilot assessments providing inputs and directions towards development of methodology for National Mental Health Survey- Mental Health Systems Assessment (NMHS-MHSA). Firstly, mental health systems and services were assessed in Kolar District of Karnataka state in India [21] by utilizing the adapted version of “World Health Organization- Assessment Instrument for Mental health Systems (WHO-AIMS)” [22]. Considering the various sources and nature of information available for assessing mental health systems at the district level, series of discussions were held with mental health programme officers and other stakeholders. Based on the feedback from stakeholders, WHO-AIMS tool was appropriately modified, finalized and piloted in Kolar district. Subsequently, district level assessment was scaled to state level wherein mental health systems was assessed in the state of Tamil-Nadu in India [23] with a specific focus on 16 districts implementing District Mental Health Program (DMHP). Learning’s from Tamil-Nadu state mental health systems assessment includes: (a) contextually modified WHO-AIMS instrument was found suitable and reliable to collate information on mental health systems at the state level, (b) required data had to be compiled from multiple sources, and (c) state level workshops and discussions with experts and stakeholders was essential for data triangulation and reaching consensus. These consensus meetings also ensured necessary buy-in of the findings by state administrators, policy makers, and program officers which is considered critical for implementing appropriate actions by the government.

### Standard operating protocol

To facilitate uniform implementation of MHSA across 12 states, standard operating protocol consisting of master protocol [24] and operational guidelines [25] document was developed and agreed upon by all team members. These documents provided step by step guide on the methodology of data collection process (what data to collect, data sources, how to collect such data as well as steps for state level expert and stakeholder consensus meeting) ensuring compilation of good quality data across states.

### Project management

**Fig. 1:**
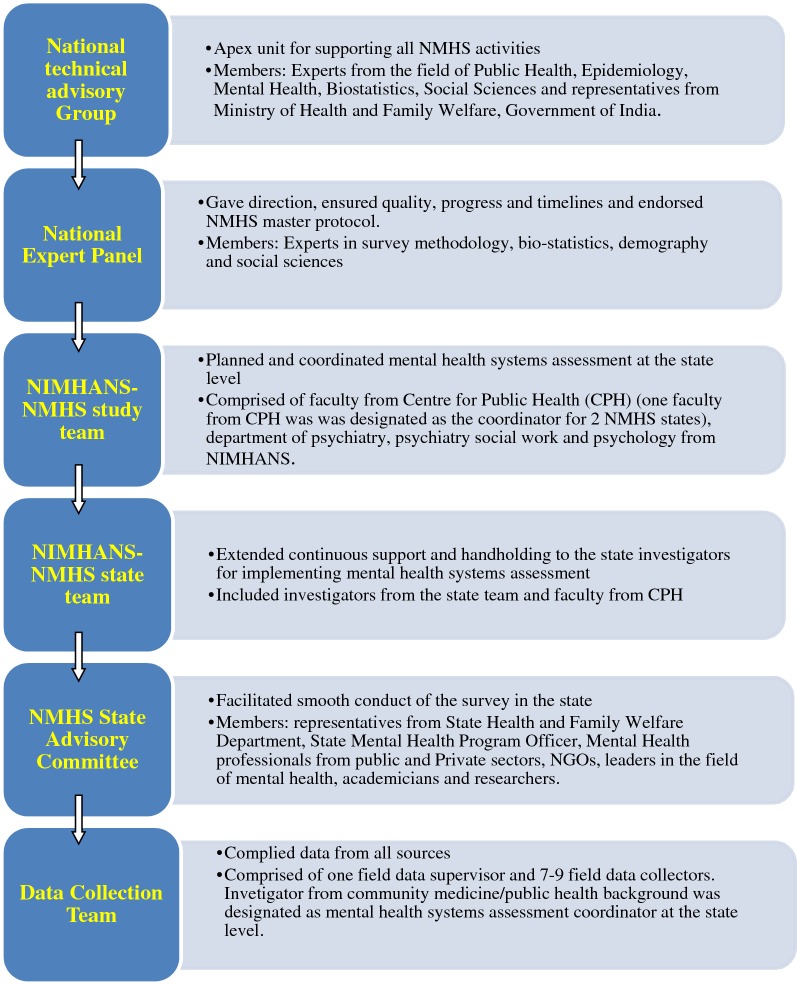
Project management–organogram of NMHS-MHSA

Since the data collection involved 12 states in India, various project management units were constituted to facilitate smooth conduct of the survey (refer Fig. 1 for organogram of NMHS-MHSA)

### Study sites

MHSA, being complimentary to NMHS, was undertaken in all NMHS surveyed states and included: Punjab and Uttar Pradesh (Northern region), Tamil Nadu and Kerala (Southern region), Jharkhand and West Bengal (Eastern region), Rajasthan and Gujarat (Western region), Madhya Pradesh and Chhattisgarh (central region) and Assam and Manipur (North-Eastern regions).

### Scope of assessment under NMHS-MHSA

The primary focus of NMHS-MHSA was overall assessment of the mental health systems at the state level. Secondly, systems assessment was undertaken in districts implementing DMHP in the NMHS surveyed states and the purpose was to review the current status of implementation of DMHP. Finally, all the other districts not implementing DMHP at the time of study were also included for assessment. The assessment at three different but interrelated levels provided comprehensive information on the status of mental health systems at the state level.

### Study instrument

The adapted version of WHO-AIMS [22] was utilized for data collection. Broadly, mental health systems was assessed under 10 different domains (refer Additional file 1: Table S1) including general information about the state, general health care facilities and resources, mental health facilities and resources, management of mental health problems, intra and intersectoral collaboration, social welfare activities, engagement with civil society, Information-Education-Communication, mental health indicators, monitoring and evaluation.

Supporting mental health programmes at the state level with relevant information for development of mental health action plan was one of the outcomes of NMHS-MHSA. Towards that and as highlighted previously, WHO-AIMS tool was appropriately adapted. Firstly, instead of focusing on detailed analysis of policy, legislation and action plan, NMHS-MHSA tool collected information on the presence and implementation status of mental health policy, legislation and action plan at the state level. Secondly, since mental health systems are evolving in the country, questions regarding human rights issues of mentally affected individuals were limited as this was observed to be difficult to assess. Thirdly, as detailed information about select parameters of mental health services (like service users categorization, number of inpatients/outpatients by different health care facilities, child and adolescent specific mental health services etc.) are routinely not available, NMHS-MHSA tool was developed to collect information on number of individuals utilizing services from mental health care facilities in the state. Fourthly, with regard to psychotropic drugs, WHO-AIMS tool collects information to estimate the proportion of health care facilities with availability of psychotropic drugs. However, the existing sources in India does not record such information and hence in NMHS-MHSA, programs managers and stakeholder’s perception regarding availability of various psychotropic drugs on a continuous and uninterrupted basis at different levels of health system (primary health center, sub-district hospital and district hospital) was collected. Finally information on mental health research is not documented in any systematic manner at the state level and hence was not considered in NMHS-MHSA.

As the development of a standardized tool that collects information to meet the specific objectives of study is of utmost importance, the tool utilized for NMHS-MHSA was validated at multiple levels. The Kolar [21] and Tamil Nadu study [23] facilitated initial validation of the NMHS-MHSA tool. Secondly, study investigators from 12 NMHS states who are experts in the field of public mental health critically examined various components of the tool and their feedback helped to further refine and validate the tool. Lastly, the tool was field tested in all the 12 NMHS states, relevant changes were made and finalized for its utilization in NMHS-MHSA.

### Data sources

The preparatory activities undertaken in Kolar [21] and Tamil Nadu [23] clearly illustrated the need to access multiple sources of data for MHSA as information was not available at one central source, either at national or state level in India. Furthermore, the field study conducted in 12 NMHS states revealed the state level variations in data sources with particular reference to health care facilities and human resources in private sector. Broadly, various data source included *national*/*state*/*district reports* (National Health Profile of India, National Rural Health Mission annual reports, Rural health statistics, District Statistical Handbook, Report on Accidental Deaths and Suicides in India by National Crime Records Bureau, Annual report by Department of Health and Family Welfare), authentic *websites* (Census of India, Medical Council of India, State Medical Council, Rehabilitation Council of India) and *personal communications with offices and individuals for collecting information from official*/*most reliable documents* (State Health and Family Welfare Department, Office of Director General of Health Services, Office of National Health Mission, District Health Office, Department of Medical Education, State Pollution Control Board, Secretary-State Mental Health Authority, State nodal officer for Mental Health, District Mental Health Programme officers, Practicing psychiatrist and psychiatric professional bodies, Civil society organizations, Mental hospitals/Institutes, Medical Colleges).

The identified data sources were approached hierarchically from the national level to the mental health facilities at the state level until adequacy and saturation were obtained for each item. An iterative process of checking and triangulating data from various sources at different levels was adopted. In situations where multiple sources of data were available for the same information, the most authentic sources were utilized.

### Training for data acquisition

NMHS-MHSA collected quantitative and qualitative information through various approaches including communication with key personnel at state level from both public and private sector. Therefore, training of study investigators and field data collectors was crucial for ensuring collection of appropriate and valid data. During national collaborators meeting, study investigators from 12 NMHS states were oriented towards the scope and nature of data to be collected, sources of data, means of resolving conflicts in case of multiple sources of data and finalizing the data set. The study investigators in turn trained the field data collectors regarding the methods, source and nature of data to be collected. The data coordinator (chosen from the team of data collectors) was given the overall responsibility of data collection and pooling in each of the study sites. Finally, continued training and handholding of the state team by the central team were part of the fortnightly e-discussions and more than 200 such e-meetings were held during the entire study period.

### Data compilation, confirmation and analysis

**Fig. 2:**
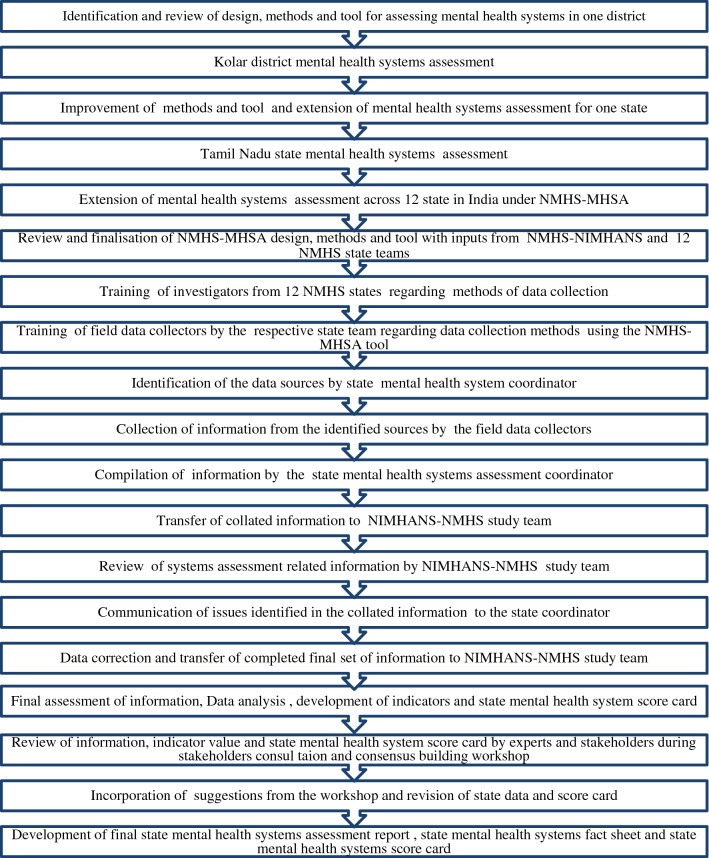
Sequential flow of activities in NMHS-MHSA

During September 2015 to March 2016, NIMHANS-NMHS team worked together with state NMHS team to compile data from various secondary sources. Subsequently, state teams forwarded the duly completed MHSA proforma to NIMHANS. Completed proforma received from all the 12 NMHS states was scrutinized by NIMHANS-NMHS team for completeness, reliability and quality of the collated information. Incomplete and inconsistent information was highlighted and communicated to the state team, following which the state team revisited the data sources and collected the appropriate information. This iterative process continued until the data was found to be complete and error free. Upon finalization of the data set, analysis was performed at the central level for all states. Frequency distributions and measures of central tendency (e.g., means, medians) were calculated as appropriate and rates per 1,00,000 population were estimated for a range of data points. Qualitative indicators were summarized through scoring system and state mental health system report card was developed. All the sequential steps beginning from preparatory activities to development of report and fact sheet sheets is depicted in Fig. 2.

### Development of indicators

Indicators to quantify the current performance and progress of mental health systems at the state level were developed based on collected data. To ensure national and international relevance of the indicators developed, key resources like WHO-mental health atlas [8] and WHO-mental health action plan [26] were reviewed and through active consultation with public health and mental health experts at state level, a set of 15 quantitative, 5 morbidity and 10 qualitative indicators were developed (refer Additional file 1: Table S2). These indicators broadly covered the following components of mental health system: availability of mental health policy, existence and functioning of state mental health authority, nature and level of implementation of mental health legislation(s), funding support for state mental health activities, presence of a mental health action plan and its implementation status, nature and level of general and mental health care facilities and human resources, training for health and non-health personnel, availability of drugs, mental health education activities, presence and mechanisms of inter-sectoral coordination, presence of monitoring and evaluation system, mental health information system, population coverage of DMHP, prevalence of mental morbidity (including prevalence of common mental disorder, severe mental disorder, alcohol use disorder, depressive disorder and high suicidal risk), incidence of suicides and treatment gap for mental disorders.

The 10 qualitative indicators essentially focused on the macro aspects of the mental health system (Additional file 1: Table S2). Each of the qualitative indicators was scored on a scale of 0–10 where 0 indicate worst and 10 indicate the best possible score and the overall score ranged between 0 and 100. The scoring was undertaken based on predetermined parameters like availability, comprehensiveness, adequacy and level of implementation depending upon the type of indicator.

### Consensus building workshop(s)

NMHS state advisory committee was formed by including group of expert’s and stakeholder’s (15 to 20 in number) in each NMHS state. The committee included: State Principal Health Secretary or their representative, State National Health Mission Director or their representative, State Mental Health Programme Officer, Member-Secretary of the State Mental Health Authority, psychiatrist from both private and public sectors, public health specialists, civil society members, legal advisors, a representative from the state Information Education Communication cell and others as required.

Following collection, compilation and analysis of data, the state mental health systems related information including the state report card was shared and discussed with the advisory committee members during the state level expert’s and stakeholder’s consultation and consensus building workshops in each of the NMHS state. Through a consultative process, the advisory committee members critically scrutinized the data and the indicators and agreed/improvised on the collated data, thereby authenticating the final information. The endorsement of the information and indicators by the advisory committee indirectly ensured its acceptance essential for follow up actions. Based on the feedback from the advisory committee members, NIMHANS-NMHS team finalized the state mental health systems assessment, related indicators and the state report card for all the 12 NMHS states.

### State mental health system report card

As a final step, each state was scored based on the final values of the 10 qualitative indicators to arrive at a composite score. The state mental health systems score along with quantitative indicators together constituted the state report card and indicate the functional status of the mental health systems at the state level. Examples of state report cards are available at (http://indianmhs.nimhans.ac.in/fact-sheets.php).

## Results

The states chosen under NMHS-MHSA were diverse with regards to their socioeconomic and administrative characteristics and are at different stages of development. The health systems especially availability of health facilities also varies across the states (Table 1). The implementation of National Mental Health Programme in India is primarily the responsibility of the state and is implemented through the District Mental Health Program (DMHP) which was launched in 4 districts in 1996 [27]. Thereafter slow and variable expansion of the program has been observed across the states. The wide variations in nature of health systems in general and mental health systems in particular provide the context for adopting the systems assessment methods described under NMHS-MHSA. The detailed results of NMHS-MHSA with regard to the performance and progress would be published subsequently.

**Table 1:** General health care facilities in NMHS states (per 1,00,000 population)

	AS	CG	GJ	JH	KL	MP	MN	PB	RJ	TN	UP	WB
Public Sector												
Population(n)	31,205,576	25,545,198	60,439,692	32,988,134	33,406,061	72,626,809	28,55,794	27,743,338	68,548,437	72,147,030	199,812,341	91,276,115
1. Super specialty Hospital	–	–	< 0.01	0.02	0.02	< 0.01	0.07	0.01	< 0.01	< 0.01	< 0.01	0.04
2. Medical college hospital	0.02	0.03	0.03	< 0.01	0.03	< 0.01	0.07	0.01	0.01	0.03	< 0.01	0.02
3. District hospital	0.08	0.09	0.05	0.07	0.11	0.06	0.24	0.08	0.05	0.04	0.07	0.02
4. Sub district/Taluka hospital	0.04	0.07	0.05	0.04	0.24	0.09	0.07	0.15	0.03	0.33	–	0.11
5. Community health centre	0.48	0.61	0.55	0.57	0.69	0.46	0.59	0.59	0.83	0.53	0.39	0.38
6. Primary health centre	3.29	3.09	1.99	1.0	2.55	1.61	2.97	1.87	3.05	2.42	1.75	0.99
7. Sub centre	14.81	20.30	12.04	11.99	16.17	12.65	14.74	10.64	21.02	12.06	10.27	11.35
8. Dispensaries	0.82	–	0.76	–	4.74	2.94	0.70	4.27	0.28	0.27	0.25	0.11
9. AYUSH hospital	0.01	0.05	0.10	0.01	0.48	0.05	0.03	0.02	0.18	0.36	0.99	0.02
10. AYUSH dispensaries	1.46	4.71	1.35	1.03	4.73	3.22	9.87	1.83	5.66	1.97	1.00	2.24
11. ESI/CGHS hospital	0.02	–	0.03	0.04	0.05	0.01	–	0.08	0.12	0.04	0.07	0.04

Health care facilities in public sector	21.03	28.93	16.96	14.80	29.82	21.14	29.37	19.55	31.22	18.08	14.82	15.32
Private sector												
1. Super specialty Hospital	< 0.01	0.01	0.02	0.02	0.07	< 0.01	1.10	0.26	< 0.01	0.02	0.02	0.02
2. Medical college hospital	0	< 0.01	0.01	0	0.06	0.01	0	0.03	< 0.01	0.03	0.01	< 0.01
3. Hospital	0.12	17.51	2.25	0.15	3.44	0.21	1.05	3.94	–	5.07	–	0.08
4. Nursing home	–	–	0.86	–	–	0.32	0.03	10.82	–	2.82	–	0.08
5. Registered clinic	–	–	0.10	–	–	1.68	1.01	2.42	–	–	–	–
6. Non allopathic hospital	–	–	–	–	–	0.28	0	0.62	–	–	–	< 0.01
*Health care facilities in private sector*	0.94	17.52	3.25	0.16	3.58	2.52	2.2	18.08	0.02	7.95	0.03	0.19
*Health care facilities in public and private sector*	21.97	46.45	20.21	14.96	33.4	23.66	31.58	37.63	31.24	26.04	14.85	15.51

Information for public health sector-India National Health Profile-2015; Information for private sector was provided by NMHS-State team. (–) indicate not known

*PB* Punjab, *UP* Uttar Pradesh, *JH* Jharkhand, *WB* West Bengal, *KL* Kerala, *TN* Tamil Nadu, *GJ* Gujarat, *RJ* Rajasthan, *AS* Assam, *MN* Manipur, *CG* Chhattisgarh, *MP* Madhya Pradesh

## Discussion

India was one of the first countries in the developing world to formulate a National Mental Health Programme as early as 1982 [28]. Based on the Bellary model [28–30], the first community mental health initiative undertaken at a district level in India, DMHP was launched in the year 1996 in 4 districts during IX Five Year Plans. Due to various implementation challenges, the program was slowly expanded across the country covering 27 districts during IX Five Year Plans (2002–2007) and 123 districts during XI Five Year Plans (2007–2012). Plans are afoot to implement DMHP across all the 624 districts in the country. In this context, previous attempts of mental health systems assessment in India primarily focused at evaluating individual components of NMHP/DMHP [13, 14, 17]. Some of the assessments also focused on quality of services especially in mental hospital/institutions and also reviewed availability of mental health human resources and facilities at the national level [12, 15, 16] and in select urban areas [31]. These studies undertaken with differing goals and objectives utilized different methodologies and tools.

NMHS-MHSA was strongly grounded on a public health approach and was undertaken considering the larger health systems assessment framework. Uniform methodology and a common data collection instrument and was employed to assess the mental health systems in 12 NMHS states at one point of time in the year 2016. Thus overcoming the limitations of the previous studies, NMHS-MHSA provides comprehensive information about the baseline status of mental health systems at the state level which would strengthen the mental health systems and program in the state and country as a whole. Simultaneous assessment of burden of mental disorders across 12 NMHS states facilitated greater understanding of the gap between need and systems response, which was a unique attempt of its kind and scale in the country.

WHO-AIMS has been widely utilized across countries for assessing mental health systems including India [8, 32]. These assessments in India usually were limited (only two state level mental health systems assessments have been conducted in India in the past) and state wide information was not available till date. Health being state subject in India [33] and with health systems and related mechanisms varying across states, there is need for information on performance of mental health systems at the individual state level [34, 35]. Therefore, NMHS-MHSA undertook assessment with a primary focus on state mental health system. Additionally, the methodology adopted also provides information at the district level required to strengthen the DMHP. With relevant changes, NMHS-MHSA tool and methodology could be utilized for repeat/periodical assessments in future and for similar assessments in India and other LAMICs.

The process of compiling information for assessing mental health systems was a major challenge in NMHS-MHSA. It is well acknowledged that all data required for such an assessment is not available at a single source or in one ministry or department as such. Hence various sources of data at multiple levels including interaction with key stakeholders were essential to gather information as well as to authenticate the same. The Kolar [21] and Tamil-Nadu [23] studies enhanced understanding of NMHS team regarding various data sources and the nature of data available in them. The type of data sources sometimes varied across the states which was apparent during the interaction with NMHS state team members and during the pilot study. Despite the challenges posed, accessing multiple data sources facilitated triangulation of data and was required to provide a clear and broad picture of mental health systems.

The baseline status of mental health systems was summarized using a set of quantitative and qualitative indicators (Additional file 1: Table S2). These indicators were developed based on the following premise: (a) reflect essential building blocks of health system, (b) feasible to collect data (c) provide actionable information and (d) facilitate national and international comparison of the mental health systems. The data required to estimate the indicator value was collated from multiple sources including interaction with key personnel. Thus there was an inherent need to authenticate the collated information which was achieved through discussion with subject experts and stakeholders during stakeholder’s consultation and consensus building workshop. This process was also vital for ensuring acceptance of the results by policy makers, program managers and all other concerned stakeholders in the state for ensuring follow up actions. Essential to highlight that comparison across states is not recommended as each of the Indian states are in different stages of development. However, it provides an opportunity to learn from strengths and limitations of individual components of a mental health programmes across surveyed states. The NMHS-MHSA also resulted in follow-up activities at the state level as seen through dissemination of data at state level, publication of state level reports, scientific communications and utilization of findings for planning and programming purposes (for details visit http://indianmhs.nimhans.ac.in/index.php).

NMHS-MHSA does have certain limitations. The absence of national registry of service providers or other such systems in the country and poorly regulated private sector posed challenges in compilation of information. Despite this challenge, the present assessment provides indicative scenario rather than accurate information on private sector. Secondly, data disaggregated by urban and rural area was not available and hence the same is reflected in NMHS-MHSA. However, often it is reported that in India there is an inequitable distribution of mental health care facilities and personnel with an urban advantage [36] and the findings from NMHS-MHSA has to be viewed on this background. Finally, the collated data could be influenced by the quality of the data sources. This is limited to the best possible extent through data triangulation and validation by experts and stakeholders during stakeholder’s consultation and consensus building workshop.

NMHS-MHSA is a rapid, reliable and comprehensive method of assessing mental health systems at provincial or state levels. Taking forward the present initiative, mental health systems assessments should be undertaken at regular intervals covering all the states in India. The tool and methodology of NMHS-MHSA is subject to continuous improvements which principally depend upon the availability of better quality data sources. Towards this end there is an absolute need for strengthening mental health information system at all levels including the mental health policy. Leveraging digital India initiative by Government of India [37], health information system in the country should be revitalized which inturn could lead to better assessments in coming years. Since NMHS-MHSA is not resource intensive and the process of data collection being robust, LMICs can utilize the methodology to assess their mental health system with contextual modifications. Mental health systems assessment undertaken at regular interval is essential for continuously guiding the national mental health policy of India [38] and also to measure and steer the country’s progress towards meeting the targets set under WHO’s mental health action plan 2013–2020 [26] and Sustainable Development Goal-3 [39].

## Supplementary information

**Additional file 1: Table S1.** Domains and components of each domain under NMHS-MHSA. **Table S2.** Mental Health Systems Assessment Indicators.

## Data Availability

The datasets used and analyzed during the current study are available from the corresponding author on reasonable request.

## Abbreviations

MSUDs: Mental and substance use disorders
DALYs: Disability adjusted life years
LMICs: Lower and middle income countries
NMHS: National Mental Health Survey
MHSA: Mental Health Systems Assessment
NMHP: National Mental Health Program
DMHP: District Mental Health Program
NIMHANS: National Institute of Mental Health and Neuro Sciences
NMHS-MHSA: National Mental Health Survey-Mental Health Systems Assessment
WHO-AIMS: World Health Organisation-Assessment Instrument for Mental Health Systems
WHO: World Health Organisation
