# Supplementary material for: Framework and approach for measuring performance and progress of mental health systems and services in India: National Mental Health Survey 2015–2016
Source: Int J Ment Health Syst. 2020 Mar 13;14:20. doi: 10.1186/s13033-020-00349-8 (PMC7071589; doi:10.1186/s13033-020-00349-8)
Supplement: Supplementary file 1 — Additional file 1: Table S1. Domains and components of each domain under NMHS-MHSA. Table S2. Mental Health Systems Assessment Indicators. [file 13033_2020_349_MOESM1_ESM.docx]

Additional file 1: Table S1: Domains and components of each domain under NMHS-MHSA

| **Domain** | **Components** |
| --- | --- |
| 1. General information | Demographic parameters of the state, number of districts, number of DMHP districts |
| 2. General health care facilities and resources | a) Number and type of general health care facilities  b) Number and type of general health human resources  c) Health information system |
| **3.** Mental health systems and resources | a) Mental health policy  b) Mental health legislations  c) Budget for mental health  d) Mental health action plan.  e) Governance (state mental health authority)  f) Mental health human resources  g) Mental health care facilities  h) Institutes for training mental health professionals  i) Mental health training of general health care personnel  j) Mental health education  k) Camp and outreach services  l) Availability of Psychotropic drugs |
| 4. Management of mental health problems | a) Patient statistics in mental hospitals  b) Involuntary admissions to mental hospitals |
| 5. Intra- and inter-sectoral collaboration | Existence and nature of collaboration with other health programs ( Reproductive and Child health programme, National Program for prevention and control of Cancer, Diabetes, Cardiovascular diseases and Stroke, National Program for Health Care of Elderly, National AIDS control Program, Revised National Tuberculosis Control Program etc) and other related sectors ( departments of education, employment, housing, social welfare, Law, police, Women and child welfare, civil society organization etc) |
| 6. Social welfare activities | Provision of disability certificates, monthly pension, reservation in jobs , preferential allotment in housing etc |
| 7. Engagement with civil society | Number, profile and nature of the services provided by NGOs for the welfare of persons with mental illness |
| 8. Information education and  communication activities | Availability of IEC materials (pamphlet, brochure, poster, video, etc.) , Use of social media for mental health awareness |
| 9. Mental health indicators | Availability and use of mental health indicators by State Health and Family Welfare department |
| 10. Monitoring and Evaluation | Process of monitoring and evaluation of various components of mental health system, availability of monitoring and or evaluation reports, Support for research by the state government |

Additional file 1: Table S2: Mental Health Systems Assessment Indicators

| **Quantitative indicators**  1. General health facilities (Public and Private sector) in the state (nos / 100000 popln)  2. Health professionals/personnel available in the state (nos / 100000 popln)  3. Districts in the state covered by DMHP (%)  4. State population covered by DMHP (%)  5. Tribal population covered by DMHP (%)  6. Mental health facilities in the state (nos / 100000 population)  7. District/General hospitals in the state providing mental health services (%)  8. Taluka hospitals in the state providing mental health services (%)  9. PHCs in the state providing mental health services (%)  10. Beds available for mental health inpatient services in the state (Nos / 100 000 popln)  11. Mental health professionals/personnel in the state (Nos / 100000 popln)  12. Health professionals/personnel in the state who have undergone training in mental  health in the last 3 years  13. Percent of total health budget allotted for mental health by state health department  14. Percentage of total allotted mental health budget that is utilized  15. Suicide incidence per 100000 population, by age and gender |
| --- |
| **Burden and treatment gap of mental morbidity**  16. Prevalence & treatment gap of Common mental disorders  17. Prevalence & treatment gap of Severe mental disorders  18. Prevalence & treatment gap of Depressive disorders  19. Prevalence & treatment gap of Alcohol use disorder  20. Prevalence & treatment gap of High Suicidal risk |
| **Qualitative indicators**  1. Mental Health Policy  2. Mental health action plan and status of its implementation  3. State mental health Co-ordination mechanism  4. Mental health budget  5. Training programme on mental health  6. Availability of Drugs  7. Availability of IEC materials and implementation of IEC activities  8. Intra and Inter-sectoral collaboration for Mental health  9. Monitoring of mental health activities  10. Implementation status of legislation pertaining to mental health |
